# Identification of Novel Peptides in Distillers’ Grains as Antioxidants, α-Glucosidase Inhibitors, and Insulin Sensitizers: In Silico and In Vitro Evaluation

**DOI:** 10.3390/nu16091279

**Published:** 2024-04-25

**Authors:** Lixin Ding, Xiuqing Zheng, Lei Zhao, Shengbao Cai

**Affiliations:** 1 Faculty of Food Science and Engineering, Kunming University of Science and Technology, Kunming 650500, China; dlx2528@163.com (L.D.); 18388417157@163.com (X.Z.); 2 Yunnan Engineering Research Center for Fruit & Vegetable Products, Kunming 650500, China; 3 International Green Food Processing Research and Development Center of Kunming City, Kunming 650500, China; 4 Beijing Engineering and Technology Research Center of Food Additives, Beijing Technology and Business University, Beijing 100048, China; zhaolei@th.btbu.edu.cn

**Keywords:** bioactive peptides, molecular docking, UHPLC-ESI-HRMS/MS, insulin resistance, reactive oxygen species

## Abstract

Distillers’ grains are rich in protein and constitute a high-quality source of various bioactive peptides. The purpose of this study is to identify novel bioactive peptides with α-glucosidase inhibitory, antioxidant, and insulin resistance-ameliorating effects from distiller’s grains protein hydrolysate. Three novel peptides (YPLPR, AFEPLR, and NDPF) showed good potential bioactivities, and the YPLPR peptide had the strongest bioactivities, whose IC_50_ values towards α-glucosidase inhibition, radical scavenging rates of 2,2′-azino-bis (3-ethylbenzothiazoline-6- sulfonic acid) (ABTS) and 2,2-diphenyl-1-picrylhydrazyl (DPPH) were about 5.31 mmol/L, 6.05 mmol/L, and 7.94 mmol/L, respectively. The glucose consumption of HepG2 cells treated with YPLPR increased significantly under insulin resistance condition. Moreover, the YPLPR peptide also had a good scavenging effect on intracellular reactive oxygen species (ROS) induced by H_2_O_2_ (the relative contents: 102.35% vs. 100%). Molecular docking results showed that these peptides could stably combine with α-glucosidase, ABTS, and DPPH free radicals, as well as related targets of the insulin signaling pathway through hydrogen bonding and van der Waals forces. This research presents a potentially valuable natural resource for reducing oxidative stress damage and regulating blood glucose in diabetes, thereby increasing the usage of distillers’ grains peptides and boosting their economic worth.

## 1. Introduction

The global incidence of type 2 diabetes mellitus (T2DM), characterized by high blood sugar levels, is steadily rising, posing a significant challenge to the worldwide healthcare system [1]. Prolonged hyperglycemia can lead to severe oxidative stress damage to blood vessels, ultimately resulting in complications such as inflammatory responses and atherosclerosis [2]. In the treatment of T2DM, the key strategies for controlling blood glucose levels involve inhibiting crucial enzymes such as α-glucosidase and improving insulin resistance status; additionally, maintaining normal levels of free radicals in the body can indirectly ameliorate diabetes complications [3,4,5]. Nowadays, common clinical hypoglycemic drugs include acarbose, voglibose, metformin, and miglitol [6,7]. While these drugs are effective in managing T2DM, they often come with certain toxic side effects and adverse reactions for long-term use of these drugs, with severe cases even leading to patient comas [8]. Hence, the pursuit of safe and effective hypoglycemic compounds and the development of high-efficiency, low-side-effect hypoglycemic functional foods have emerged as a new research focus.

Food-derived bioactive peptides are generally low-molecular-weight compounds that are safe, low in toxicity, and easily absorbed and that exhibit good antibacterial, antihypertensive, hypoglycemic, immunomodulatory, and antioxidant properties [9,10,11]. Since these peptides have small molecular weights, they are more likely to be absorbed intact by the gastrointestinal tract after oral ingestion, thereby exerting systemic or local effects [12]. Consequently, the study of bioactive peptides has progressively emerged as a prominent research area in recent years. Numerous studies have demonstrated that certain bioactive peptides can regulate blood glucose levels and enhance the management of type 2 diabetes through diverse pathways, such as the inhibition of α-glucosidase activity or the improvement of insulin resistance [13,14]. For instance, Mora et al. (2020) identified the peptides AEEEYPDL and LGVGG from Spanish dry-cured ham, which effectively inhibited α-glucosidase activity [13]. In the study conducted by Hu et al. (2019), water-extracted peptides and proteins were found to alleviate insulin resistance, thereby improving diabetes by enhancing glucose utilization [14].

Distillers’ grains, by-products of rice wine production, possess abundant protein and alimentary value. Nevertheless, currently, most distillers’ grains are primarily utilized as animal feed, resulting in potential resource wastage. Hence, the purpose of this study is to identify novel bioactive peptides with good α-glucosidase inhibition, antioxidant capacity, and insulin resistance-ameliorating effects from distiller’s grains protein hydrolysate and to further reveal the underlying mechanisms. This research may provide new insights into the use of distillers’ grains peptides as a valuable natural resource to combat oxidative stress damage and regulate blood glucose levels in diabetes, and the outcomes may hold the potential for enhancing the economic benefits derived from distillers’ grains peptides.

## 2. Materials and Methods

### 2.1. Chemical and Reagents

Distillers’ grains were generously supplied by the Suzhou Bridge Wine & Spirits Co. (Suzhou, China). Peptides YPLPR, AFEPLR, and NDPF (purity ≥ 98%) were composed by All Peptide Biotechnology Co., Ltd. (Hangzhou, China). Dulbecco’s Modified Eagle Medium (DMEM) medium, embracing 10.0% fetal bovine serum (FBS) and 1.0 mol/L phosphate buffer (PBS) was bought from Gibco (Life Technologies, Inc., Carlsbad, CA, USA). Alkaline protease (2.4 U/g), penicillin-streptomycin (100.0 U/mL penicillin-100.0 mg/mL streptomycin), 3-(4,5-dimethylthiazol-2)-2,5-diphenyltetrazolium bromide (MTT) powder, 2′,7′-dichlorofluorescence yellow diacetate (DCFH-DA), and trypsin solution were purchased from Solarbio (Beijing Solarbio Science and Technology Co., Ltd., Beijing, China). The HepG2 cell line was acquired from the Kunming Institute of Animal Science, Chinese Academy of Sciences (Kunming, China). Rosiglitazone, insulin, ABTS, DPPH, α-glucosidase derived from *Saccharomyces cerevisiae* (type I, protein content of at least 30.0 units per milligram), and p-nitrobenzene α-D-glucopyranoside (pNPG, with a minimum purity of 99.0%) were purchased from Sigma-Aldrich (located in Shanghai, China). The glucose kit was purchased from Nanjing Jiancheng Co. (Nanjinng, China). Other reagents applied were all of analytical rating.

### 2.2. Sample Preparation

Distillers’ grains underwent lyophilization using the Christ Alpha 1-2 LD plus lyophilizer and then were finely made into a powder using the Lingdan LD-T300 grinder. The isolation of protein from the distillers’ grains followed the way outlined by Wang et al. (2019) (minor modifications) [15,16]. The concrete method of separating proteins is displayed in the Supplementary Materials.

### 2.3. Composition Analysis of Peptide by UHPLC-ESI-HRMS/MS

The molecular mass and amino acid identity of peptides in rice protein hydrolysate were determined by the Thermo Fisher Ultimate 3000 UHPLC system (Thermo Fisher Scientific, Dreieich, Germany) combined with the Agilent Zorbax SB-C18 column (1.7 μm, 2.1 mm × 100 mm, Dr. Asp 80Maisch, Germany). According to the Tian et al. (2022) method (slightly modified), the mass spectrometry parameters were set [17]. Details of the method are shown in the Supplementary Materials. Peak Studio 8.0 (Bioinformatics Solutions, Waterloo, ON, Canada) was used to analyze molecular mass and amino acid sequence. Peptides with an average local confidence (ALC) score exceeding 85% were deemed to be included.

### 2.4. Determination of α-Glucosidase Inhibitory Effect

The inhibitory effect on α-glucosidase activity of the protein hydrolysates from distillers’ grains or three selected peptides was assessed following the methodology outlined by Zhang et al. (2018) with slight modifications, and the concrete method is shown in the Supplementary Materials [18].

### 2.5. Assessment of Scavenging Capacity against DPPH and ABTS Radicals

The antioxidant properties of vinasse hydrolysates or three screening peptides were evaluated by DPPH and ABTS free radical scavenging experiments according to the method of Sun et al. (2015) [19]. Detailed information about the test method has been recorded in the Supplementary Materials.

### 2.6. In Silico Screening of Main Bioactive Peptides in Distillers’ Grains Protein Hydrolysates

Peptide bioactivity prediction was conducted using Peptide Ranker (http://distilldeep.ucd.ie/PeptideRanker/, (accessed on 12 October 2023)) and selected peptides with a biological activity score of ≥0.6 for further analysis. It is important to highlight that a higher score indicates a higher likelihood of bioactivity [17]. The detailed method of studying the interaction between distiller’s grains protein hydrolysate polypeptide and α-glucosidase by molecular docking technology is noted in the Supplementary Materials. For the pretreatment of peptides, refer to O’Boyle et al. (2011) [20]. Given the unavailability of the 3D structure of α-glucosidase, Saccharomyces cerevisiae isomaltose (PDB Code: 3A4A) from the database RCSB PDB (http://www.rcsb.org/pdb/home/home.do, (accessed on 12 October 2023)) was employed as a substitute. The post-treatment of α-glucosidase and the subsequent molecular docking followed the approach [10]. Based on the results of the molecular docking, peptides with an affinity equal to or superior to WLRL were identified as potential α-glucosidase inhibitory peptides [14]. The structure and interactions of latent α-glucosidase inhibitory peptides were analyzed using PyMOL (version 2.3.1). ToxinPred (http://crdd.osdd.net/raghava/toxinpred/ (accessed on 25 October 2023)) was used to appraise the toxicity of peptides [21]. Additionally, the solubility of peptides was evaluated by an online website (http://www.innovagen.com/proteomicstools, (accessed on 26 October 2023)) [22].

### 2.7. Peptides Synthesis and Activity Verification

Peptides YPLPR, AFEPLR, and NDPF, derived from distillers’ grains protein hydrolysates, were synthesized. The synthesized peptides were stored, and their corresponding activities were verified through in vitro experiments. The α-glucosidase inhibitory effects and free radical scavenging capacities were performed as described above in Section 2.4 and Section 2.5.

### 2.8. Determination of Insulin-Resistance HepG2 Cells on Bioactive Peptides

The MTT method was adopted to assess the impact of samples and insulin on cell viability, helping determine the optimal concentrations of insulin and the samples. The experimental protocol for improving insulin resistance followed previous methods [23,24]. Details are provided in the Supplementary Materials. In this experiment, various groups were established, incorporating the blank control group (K), model group (M), peptide sample groups, and the positive control group (treated with rosiglitazone).

### 2.9. Evaluation of Scavenging Capacity against Intracellular ROS

According to Liu et al. (2020), the H_2_O_2_-induced scavenging activity of reactive oxygen species was evaluated [25]. In this experiment, various groups were established, including the blank, sample, positive control, and model groups. Refer to the Supplementary Materials for specific information.

### 2.10. Molecular Docking to Probe Mechanism of Action

The molecular docking of the peptides YPLPR, AFEPLR, and NDPF with ABTS and DPPH was performed in a manner consistent with Section 2.6. The 3D structures of ABTS (Code: 5360881) and DPPH (Code: 2735032) were obtained from PubChem (https://pubchem.ncbi.nlm.nih.gov/ (accessed on 16 November 2023)). The phosphorylated insulin receptor tyrosine kinase protein (PIRTK protein) and peroxisome proliferator-activated receptor γ (PPARγ) were important targets involved in the insulin signaling pathway [26,27]. The 3D structures of the PIRTK protein (PDB Code: 1IR3) and PPARγ (PDB Code: 2PRG) were obtained from the RCSB PDB database. The grid boxes and center of 1IR3 were 80 × 80 × 80 Å and −24.099, 39.015, 11.431 (X, Y, Z) [26]. The grid boxes of 2PRG had 80 × 80 × 80 Å and grid centers of 50.140, −38.202, and 19.559 (X, Y, Z) [27]. Then, the peptides YPLPR, AFEPLR, and NDPF were docked with proteins. Their results were visualized by PyMOL (version 2.3.1).

### 2.11. Statistical Analysis

All experiments were conducted at least three times. The data were disposed of and analyzed by Origin 2021 (Origin Lab, Northampton, MA, USA) and SPSS 26 (Chicago, IL, USA) software. The data were represented by a median of 25% and a 75% quartile, and significance was determined by the Kruskal–Wallis test (*p* < 0.05).

## 3. Results and Discussion

### 3.1. Identification of the Peptides in Distillers’ Grains Protein Hydrolysates

In the process of hydrolysis, the degree of hydrolysis of distiller’s grains protein gradually augmented with the increase in time, and after hydrolysis for 2.5 h, the degree of hydrolysis gradually remained steady, indicating that the enzymatic reaction also stabilized. The final hydrolysis degree of alkaline protease was 18.49 ± 0.49%. In previous studies, the final hydrolysis degree of alkaline protease for soybean protein was 19.00% [28], and that for mung bean protein was about 25.00% [29]. In the lump, 20 peptides with ALC scores > 85% were identified among the distillers’ grains protein hydrolysate peptides (Table 1). These 20 peptides contained 4–8 amino acids, and their molecular weights ranged from 438–860 Da; all of them were low-molecular-weight peptides. The study of beer and spent grain peptides also showed that the main bioactive peptides were low-molecular-weight peptides with relative molecular masses < 1500 Da [11,17]. The better bioactivity of low molecular mass peptides is mainly due to the fact that they can bind more easily to the active sites of specific enzymes or be more readily absorbed by the gastrointestinal digestive tract and blood vessel system and eventually demonstrate physiological regulatory properties, which also shows that distillers’ grains protein hydrolysate peptides may have good biological activity. And in previous studies on rice wine, 99 peptides with ALC scores > 85% were also identified from rice wine [10]. The number of peptides in rice wine was higher than that in distillers’ grains protein hydrolysates, mainly because many peptides were further broken down into more peptides during the fermentation of rice wine. The biological activity of distillers’ grains protein hydrolysate peptides needs to be further explored and developed.

### 3.2. α-Glucosidase Inhibitory Effect of Distillers’ Grains Protein Hydrolysates

**Figure 1:**
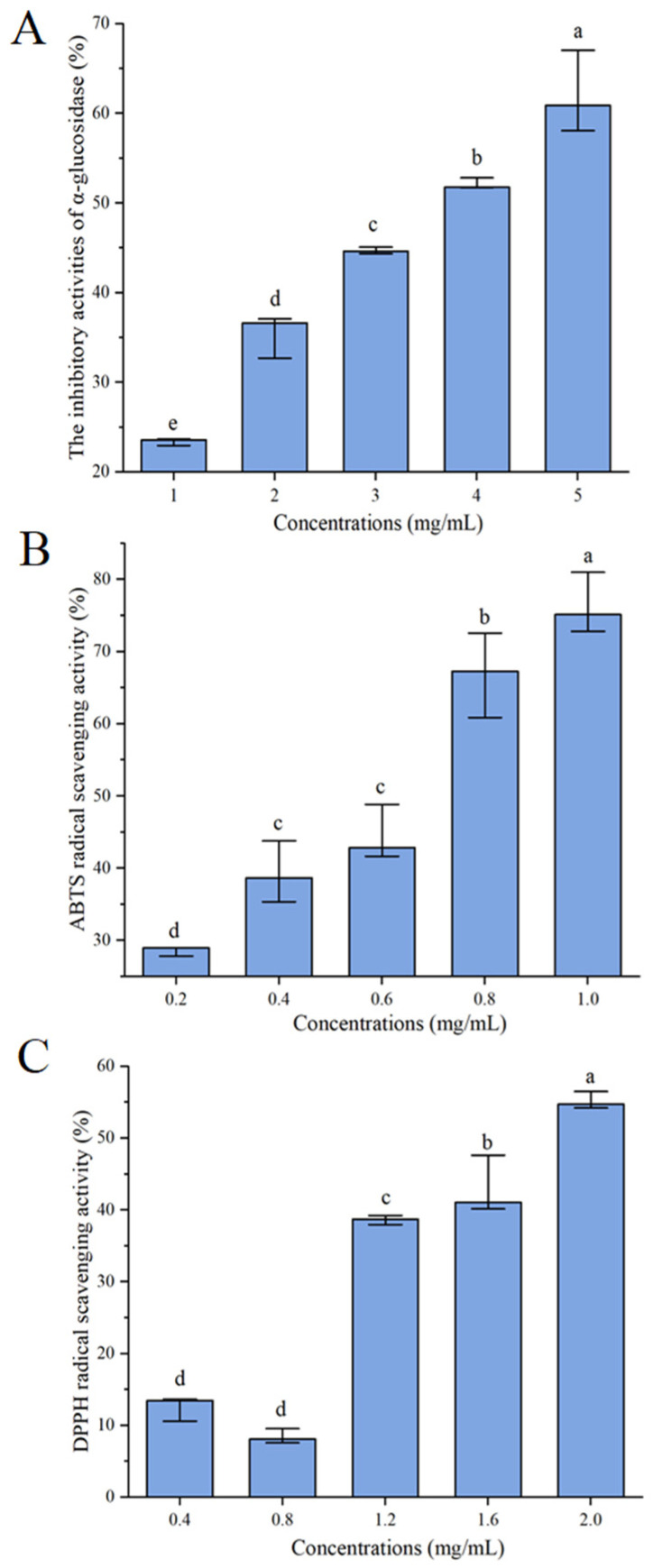
α-Glucosidase inhibitory activities (**A**), ABTS (**B**), and DPPH (**C**) scavenging activities of protein hydrolysate from distillers’ grains. The data are displayed as the median (n = 3) with the 25% and 75% quartiles and analyzed using the Kruskal–Wallis test. Significance is represented by different letters (*p* < 0.05).

α-glucosidase is a membrane-associated enzyme. Inhibition of α-glucosidase activity can reduce carbohydrate hydrolysis, thus reducing sugar absorption and effectively controlling diabetes. [11]. As shown in Figure 1A, with the increase in concentration, the α-glucosidase inhibitive activity of distiller’s grains protein hydrolysate increased, and the α-glucosidase inhibitive activity was 60.88 (58.07, 67.04) % when the concentration of distillers’ grains protein hydrolysates was 5 mg/mL, with an IC_50_ value of 3.76 (3.51, 3.83) mg/mL. In the study on rice wine peptides, the α-glucosidase inhibitory activity of 50% ethanol-eluted rice wine peptides had an IC_50_ value of 1.71 (1.65, 1.74) mg/mL [10]. And the IC_50_ value of α-glucosidase depressant activity was 4.94 ± 0.07 mg/mL in the study of soy protein hydrolyzed peptide [15]. The comparison indicated that the distillers’ grains protein hydrolysates had α-glucosidase inhibitive activity and deserves more deep investigation and development.

### 3.3. Scavenging Capacity against DPPH and ABTS Radicals of Distillers’ Grains Protein Hydrolysates

The oxidation stress is nearly relevant to diabetes. Reducing the production of free radicals, or directly breaking the free radicals produced by the body, can reverse the tissue damage caused by oxidative stress, thus preventing or delaying diabetes complications. While ABTS and DPPH free radicals are the most common free radicals in the body, the scavenging activities of distillers’ grains protein hydrolysates on ABTS and DPPH free radicals are shown in Figure 1B,C. The scavenging activity of distillers’ grains protein hydrolysates on ABTS and DPPH radicals increased gradually with increasing concentrations, with IC_50_ values of 0.59 (0.52, 0.64) mg/mL and 1.85 (1.74, 1.86) mg/mL, respectively. This shows that distillers’ grains protein hydrolysates have good antioxidant activity.

### 3.4. Screening of Main Bioactive Peptides in Distillers’ Grains Protein Hydrolysates

Peptide Ranker (http://distilldeep.ucd.ie/PeptideRanker/ (accessed on 12 October 2023)) was used to score the probability of biological activity for each peptide sequence, and the outcomes are shown in Table 2. The closer the probability of a peptide being biologically active predicted by Peptide Ranker to 1, the more confident Peptide Ranker is that the peptide is biologically active. Peptides with a score above or equal to 0.6 were more likely to be biologically active [16,17]. Based on the scoring results, ten peptides with scores greater than 0.6 were selected, which were RFDR, PDVGHPM, YPLPR, AFEPLR, FEPLR, WLDY, FEPL, FDGVLRGP, NDPF, and WNLN.

Due to the fact that α-glucosidase is a more important and directly regulated target in the treatment of diabetes, the potential inhibitory activity of α-glucosidase was used as the main condition of inquiry for further analysis. According to a previous study, the WLRL peptide from soybean protein exhibited a good inhibitory effect toward α-glucosidase [15]. Therefore, this peptide was selected as a positive control to facilitate the analysis of the current study, and its affinity grading after molecular docking with α-glucosidase was −9.1 kcal/mol. Four peptides, YPLPR, AFEPLR, NDPF, and WNLN, had affinities of −10.7, −9.6, −9.1, and −9.2 kcal/mol, respectively, which were better than WLRL, indicating that these peptides may have good α-glucosidase inhibitory potential. Therefore, these peptides can be used as potential functional candidates for α-glucosidase inhibitors for further analysis.

The solubility and toxicity of bioactive peptides are closely related to the biological activity of the peptide. Before further application, it plays a large part in analyzing the toxicity of peptides. The Toxin Pred tool is used to predict whether or not peptides are toxic, and it is being utilized more widely to assess numerous bioactive peptides [30]. The prediction results obtained using this tool showed that all the peptides screened in this study were safe and non-toxic. The potential efficacy of bioactive peptides is closely related to the solubility of the peptides, which was assessed by the peptide property calculator Innovagen. The results are shown in Table 2. Among the four peptides obtained by bioactivity scoring, α-glucosidase inhibition binding energy, and toxicity prediction, YPLPR, AFEPLR, and NDPF had good solubility. Therefore, YPLPR, AFEPLR, and NDPF were selected as the main potentially bioactive peptides for further study.

### 3.5. Activity Verification of Synthetic Novel Peptides

YPLPR had the best inhibitory effect with an IC_50_ value of 5.31 (5.28, 5.35) mmol/L; NDPF had the second inhibitory effect, and AFEPLR had the worst, with IC_50_ values of 6.24 (5.97, 6.35) and 8.69 (8.67, 8.71) mmol/L. These studies have previously identified bioactive peptides with α-glucosidase depressant activity in foods. The peptides LDLQR, AGGFR, and LDNFR from malt were found to exhibit significant α-glucosidase inhibitory activity, with IC_50_ values of 8.59 ± 0.25, 8.66 ± 0.33, and 9.21 ± 0.22 mmol/L, respectively [31]. In contrast, in previous studies on rice wine, α-glucosidase with peptides SSLFR, QFTPR, and FTYPR depressant activity was also discovered, and their IC_50_ values were 7.73 (7.73, 7.74), 9.18 (9.17. 9.18), and 3.57 (3.26, 3.68) mmol/L, respectively [11]. The results of this study, compared with previous studies, all indicated that YPLPR, AFEPLR, and NDPF had good α-glucosidase depressant activity.

The IC_50_ values of ABTS radical scavenging for YPLPR, AFEPLR, and NDPF were 6.05 (5.9, 6.64), >15, and >15 mmol/L, respectively, and the scavenging of DPPH radicals was better than that of ABTS, with IC_50_ values of 7.94 (7.92, 7.96), 13.80 (13.72, 13.94), and 8.71 (8.69, 8.74) mmol/L, respectively. The peptides EAMAPK and AVPYPQ, identified in the research of Pepe et al. (2016), showed antioxidant activity at different concentrations (5–150 mg/mL) [32]. The peptide FTYPR, previously screened from rice wine peptides by molecular simulation, had IC_50_ values of 2.64 (2.63, 2.66) and 6.09 (6.04, 6.82) mmol/L for ABTS radical and DPPH radical scavenging capacity, respectively. It is thus evident that YPLPR, AFEPLR, and NDPF have antioxidant activity; YPLPR has the best antioxidant activity and can be further developed and processed as a potential functional food.

### 3.6. Improvement of Bioactive Peptides on Insulin Resistance in HepG2 Cells

**Figure 2:**
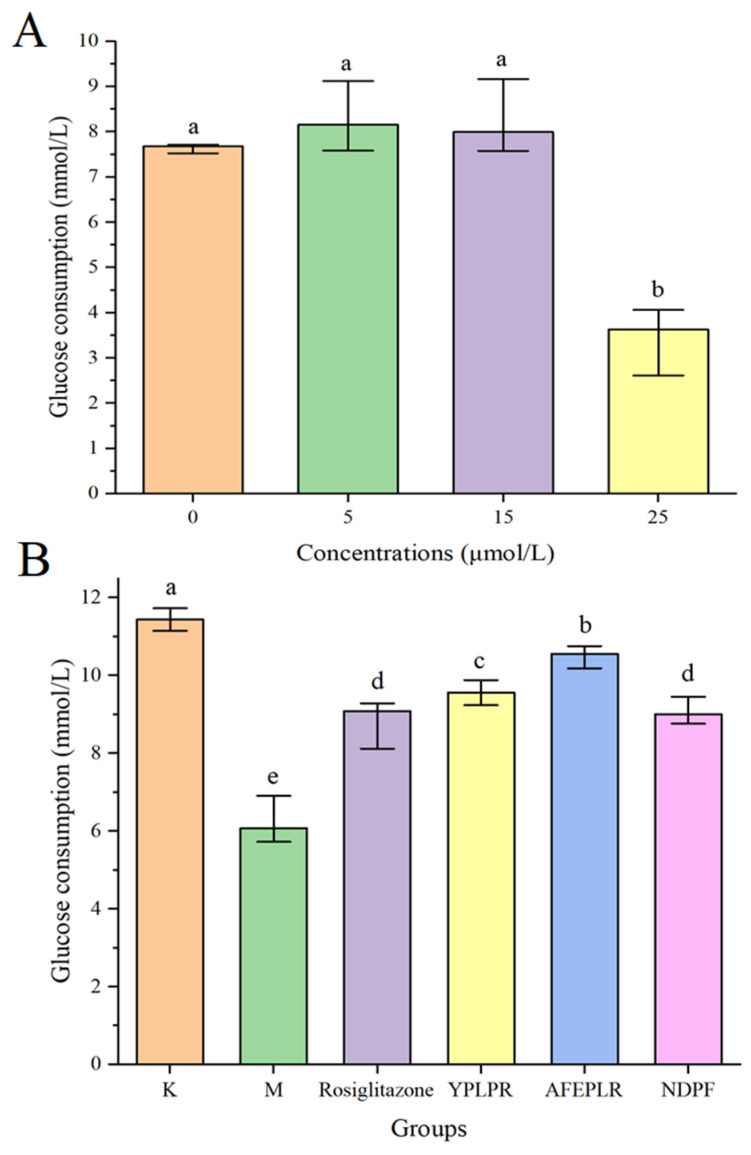
The effects of different concentrations of insulin on glucose consumption of HepG2 cells (**A**); the effects of YPLPR, AFEPLR, NDPF, and rosiglitazone on glucose consumption of HepG2 cells (**B**). Data are presented as the median (n = 6) with the 25% and 75% quartiles; different letters indicated significant differences (*p* < 0.05). (K: control group; M: model group; rosiglitazone: positive control group).

Insulin is one of the main pathogenic mechanisms of type 2 diabetes mellitus, and the liver is an important site for insulin to function. HepG2 cells are derived from human embryonic hepatocytes and possess many biological characteristics of normal hepatocytes, so HepG2 cells are chosen to establish an insulin resistance model. As the cellular insulin resistance condition arises, the utilization of glucose by the cells decreases, and the consumption of glucose decreases. Therefore, insulin resistance is usually expressed by the consumption of glucose in studies [24]. Using the MTT method, this study explored the effect of different concentrations of insulin on cellular glucose consumption after 24 h of action with HepG2 cells. As shown in Figure 2A, the glucose consumption of the cells gradually decreased as the concentration of insulin increased. When the insulin concentration reached 25.0 µmol/L, the cellular glucose consumption was significantly reduced compared with the group without insulin, which indicated that HepG2 cells developed insulin resistance. And MTT experiments showed that insulin was not cytotoxic at a concentration of 25.0 µmol/L. Therefore, the insulin concentration of 25.0 µmol/L was chosen to establish the insulin resistance model in this experiment. Also, MTT results showed that YPLPR, AFEPLR, and NDPF had no cytotoxicity at 1.0 mmol/mL, so the sample concentrations for subsequent experiments were all 1.0 mmol/L.

In this study, the effect of YPLPR, AFEPLR, and NDPF on the glucose consumption of HepG2 cells in an insulin resistance state was investigated. As shown in Figure 2B, the glucose consumption of model group M was 6.08 (5.80, 6.81) mmol/L, which was significantly lower than that of blank control group K (*p* < 0.05), indicating that the insulin resistance model of this experiment was successful and the results of the relevant experiments were obtained under the condition that the cells were insulin resistant. The glucose consumption of peptides YPLPR, AFEPLR, and NDPF were 9.56 (9.37, 9.83), 10.56 (10.21, 10.71) and 9.00 (8.80, 9.33) mmol/L, respectively. The glucose consumption of these three peptides was significantly increased compared with model group M; YPLPR and AFEPLR were significantly different from the positive control rosiglitazone group (9.09 (8.29, 9.23) mmol/L) (*p* < 0.05). The efficacy of food-derived peptides to improve insulin resistance has been studied previously, and in a study of walnut-derived peptides, the identified peptides LVRL, LRYL, and VLLALVLLR all increased glucose consumption in HepG2 cells compared to the model group and served to improve insulin resistance [33]. In a study by Gong et al. (2020), specific bioactive peptides SDIPNPIGSE, NPWDQVKR, and QEPVLGPVRGPFP extracted from goat milk casein hydrolysate have demonstrated an improvement in insulin resistance in HepG2 cells exposed to high glucose. This study establishes the potential of goat cheese protein hydrolysate as a food component for enhancing insulin sensitivity [34]. Comparison with these studies indicates that the peptides YPLPR, AFEPLR, and NDPF have strong effects on improving insulin resistance, all of which can promote cellular glucose uptake, and these peptides have some development and utilization value.

### 3.7. Intracellular ROS Scavenging Activity by Bioactive Peptides

**Figure 3:**
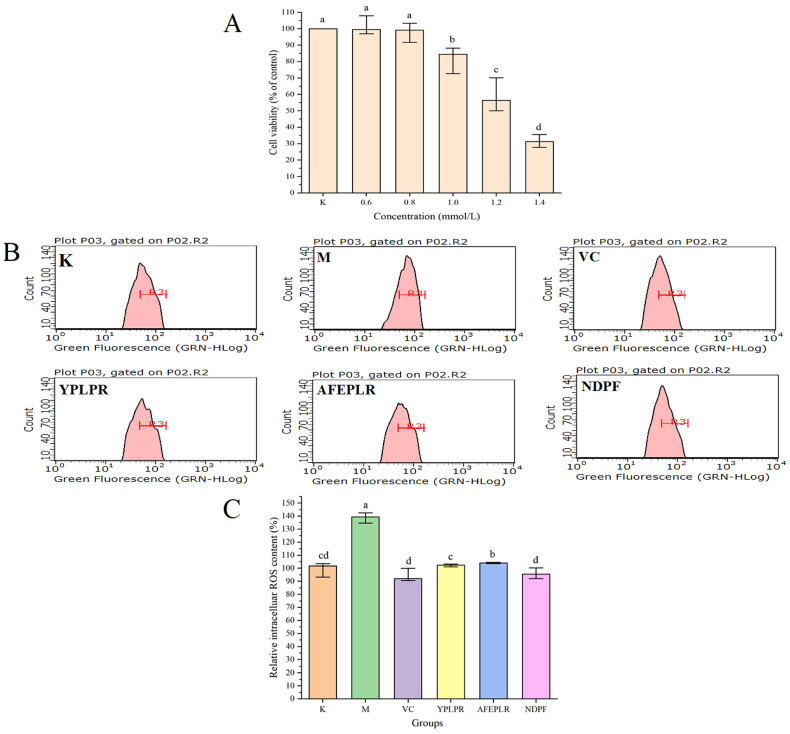
Scavenging of H_2_O_2_-induced intracellular ROS production by YPLPR, AFEPLR, NDPF, and VC. Effects of different concentrations (**A**) on cell viability; ROS flow chart, R3 represents half the peak width of the blank group K. (**B**); histogram of ROS contents (**C**). Data are displayed as the median (n = 4) with the 25% and 75% quartiles; different letters indicated significant differences (*p* < 0.05). (K: control group; M: model group; VC: positive control group).

Excessive ROS may attack normal cells or biological macromolecules and undergo oxidative stress, causing severe damage to cellular structures, which causes many diabetic complications and chronic diseases. Accordingly, in this study, the scavenging activities of ABTS and DPPH radicals of bioactive peptides were not only investigated by in vitro experiments, but their antioxidant activity was also further investigated at the cellular level. The cell damage rate of the H_2_O_2_-induced cell damage model was generally around 50%. As shown in Figure 3A, with the increase in concentration, the cytotoxicity produced by H_2_O_2_ was enhanced, and the cell viability gradually decreased. When the concentration of H_2_O_2_ was 1.2 mmol/L, the cell viability rate was 56.38 (50.33, 68.00) %, so the concentration of H_2_O_2_ of 1.2 mmol/L established the cell damage model in this study. And the intracellular ROS levels in the model group established by 1.2 mmol/L H_2_O_2_ were significantly higher than those in the blank group (*p* < 0.05), indicating that the oxidative stress model of HepG2 cells induced by 1.2 mmol/L H_2_O_2_ was successfully established. Consistent with 3.6, YPLPR, AFEPLR, and NDPF were used at 1.0 mmol/L for subsequent experiments.

The intracellular ROS inhibition of the different fractions was represented by flow and histograms. In the flow diagram (Figure 3B), the left-right shift of the peak indicates the magnitude of the fluorescence luminosity, and the more the peak is shifted to the right, the greater the fluorescence intensity. The peak of the model group M was significantly shifted to the right compared with the blank group K, which indicated an increase in fluorescence intensity and an increase in ROS content. As shown in Figure 3C, the relative intracellular ROS content in the M group and the blank group K were 139.46 (135.10, 142.41) % and 101 (95.10, 103.50) %, respectively. The relative intracellular ROS content in the M group was significantly higher than that in the blank group K (*p* < 0.05), which indicated that the oxidative stress model in this batch of experiments was successful. The relative intracellular ROS content in VC, the YPLPR, AFEPLR, and NDPF groups were 92.03 (90.83, 98.11) %, 102.35 (101.38, 103.06) %, 104.11 (103.69, 104.54) % and 95.47 (92.68, 99.37) %, respectively. It can be seen that the intracellular ROS content in the YPLPR, AFEPLR, and NDPF groups was relatively lower than that in the M group; among them, the YPLPR and NDPF intracellular ROS contents were not significantly different from that in the VC group and the blank group (*p* > 0.05). This indicated that the peptides YPLPR, AFEPLR, and NDPF played a good protective role against oxidative stress damage to cells and had a good scavenging effect on reactive oxygen species.

### 3.8. Molecular Docking

#### 3.8.1. The Molecular Docking of Bioactive Peptides with α-Glucosidase

**Figure 4:**
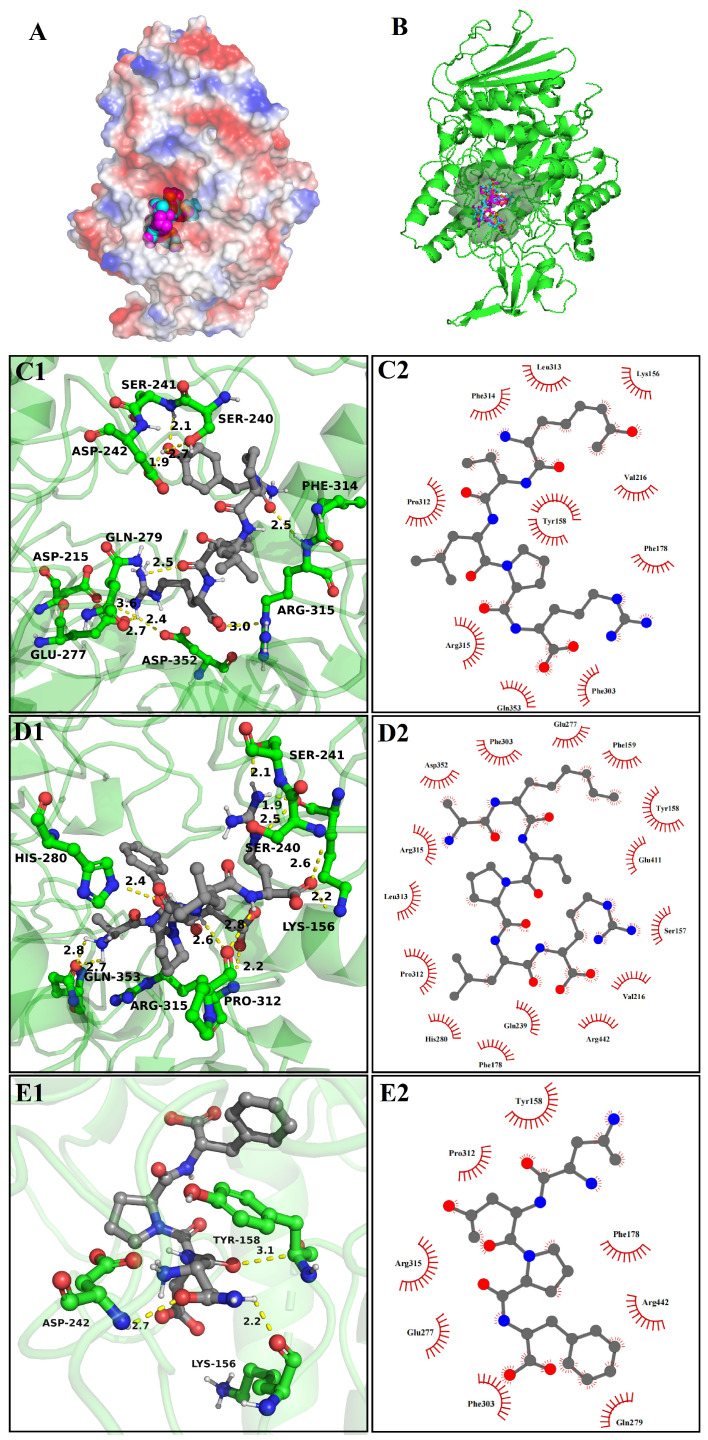
Molecular docking results of YPLPR, AFEPLR, and NDPF with α-glucosidase (PDB Code: 3A4A). The 3D structural surface images of three peptide with α-glucosidase (**A**); active site binding of three peptide with α-glucosidase, (**B**), where YPLPR is in blue, AFEPLR in purple and NDPF in red. The 3D images of the hydrogen bonding of YPLPR (**C1**), AFEPLR (**D1**), and NDPF (**E1**) with the amino acid residues of α-glucosidase (The yellow dotted line represents the hydrogen bond distance, green part is α-glucosidase, and gray part is peptide.), and the 2D images of the hydrophobic interaction of YPLPR (**C2**), AFEPLR (**D2**), and NDPF (**E2**) with the amino acid residues of α-glucosidase.

To reveal the possible inhibition mechanisms of YPLPR, AFEPLR, and NDPF towards α-glucosidase, the optimal docking postures between α-glucosidase and three peptides were shown in Figure 4, and the related results are shown in Table 3. Usually, when α-glucosidase binds to the ligand, the active cavity provides a strong hydrophobic environment and multiple hydrogen bond binding sites, which are conducive to the steadiness of the ligand. As shown in Figure 4A,B, YPLPR, AFEPLR, and NDPF can bind to amino acids in the active lumen of α-glucosidase and be completely encapsulated in the α-glucosidase active pocket, forming a relatively stable binding conformation. It has been shown that hydrogen bonding and hydrophobic interactions are important indicators of ligand-receptor binding. Among them, the number and distance of hydrogen bonds are the main drivers of docking ligand–α-glucosidase interaction, where the ligand inhibits α-glucosidase activity by binding to α-glucosidase active amino acids through hydrogen bonds and preventing substrate access to the active pocket [35]. As shown in Figure 4C1,C2 and Table 3, YPLPR formed hydrogen bonds with 9 amino acid residues (Asp215, Ser240, Ser241, Asp242, Glu277, Gln279, Phe314, Arg315, and Asp352) in the α-glucosidase active pocket, and the longest of these bonds was 3.6 Å, the shortest was 1.9 Å. As shown in Figure 4D1,D2 and Table 3, AFEPLR established 11 hydrogen bonds with 7 amino acid residues in the active site of α-glucosidase, with a mean bond distance of 2.4 Å. And it also had hydrophobic interactions with 15 amino acids on α-glucosidase. In contrast, NDPF formed only 3 hydrogen bonds with Lys156, Tyr158, and Asp242 on α-glucosidase, with bond lengths of 2.2 Å, 3.1 Å, and 2.7 Å, respectively. And it had hydrophobic interactions with 8 amino acid residues on α-glucosidase (Figure 4E1,E2). The results indicated that YPLPR, AFEPLR, and NDPF were able to efficiently attach to the active site of α-glucosidase. In previous studies, Asp69, Tyr158, Asp215, Val216, Ser240, Asp242, Arg442, Gln279, Alp411, and Asn415 were identified as the primary active residues responsible for α-glucosidase inhibition [36]. The comprehensive analysis shows that YPLPR interacts with the key residues that inhibit α-glucosidase activity the most, namely Asp215, Ser240, Asp242, and Gln279, which is consistent with the highest YPLPR and α-glucosidase binding scores and the best actual α-glucosidase inhibition experimental activity (3.5 Experimental Results). This result indicates that computer simulation is desirable to be used to assist experimental studies, and it can greatly reduce the cost and time of traditional biological screening, making it an effective method for screening enzyme-inhibiting peptides.

#### 3.8.2. The Molecular Docking of Bioactive Peptides with ABTS and DPPH

**Figure 5:**
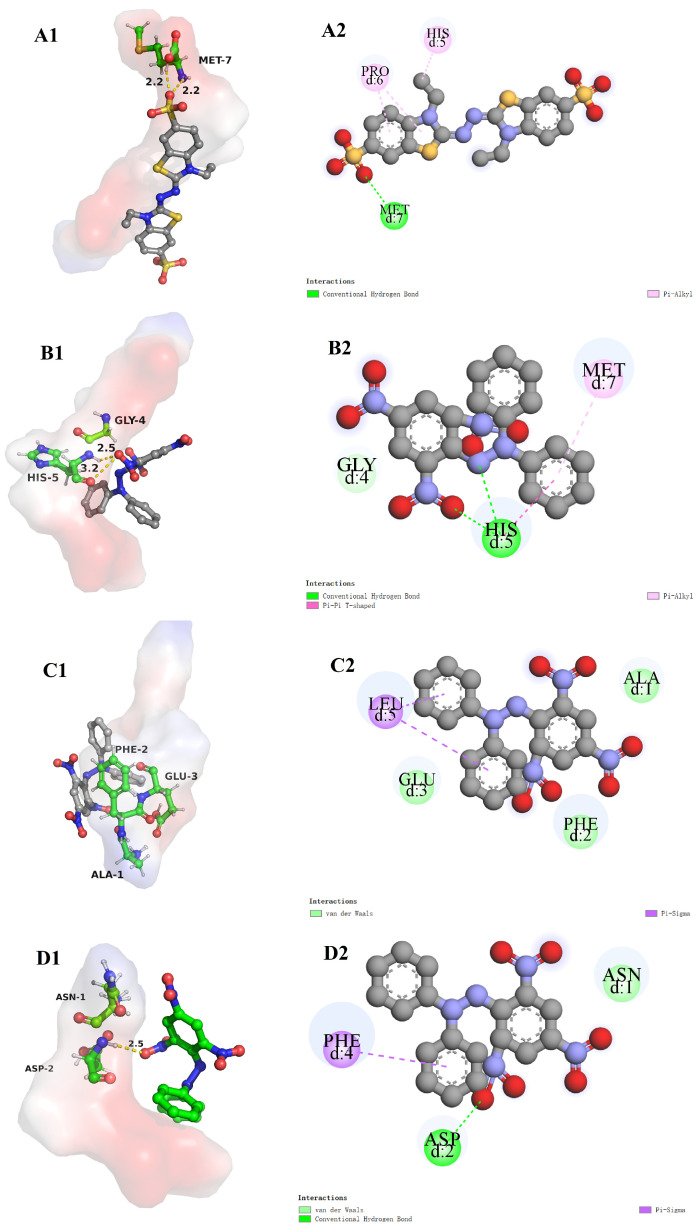
The molecular docking results of YPLPR (**A1**,**A2**) with ABTS and YPLPR (**B1**,**B2**), AFEPLR (**C1**,**C2**), and NDPF (**D1**,**D2**) with DPPH. Peptides are displayed by surface style, ABTS, and DPPH are displayed by rod and ball model. The yellow dotted line represents the hydrogen bond distance.

The radical scavenging mechanisms of the three peptides YPLPR, AFEPLR, and NDPF on ABTS and DPPH radicals were also investigated by molecular docking. As shown in Figure 5A1,A2, the binding energy of YPLPR to the ABTS radical is −4.2 kcal/mol. ABTS, as a ligand, can form 2 hydrogen bonds with Met7 and Pi bonds with His5 and Pro6 on YPLPR. As shown in Figure 5B1–D2, the binding energies of YPLPR, AFEPLR, and NDPF to the DPPH radical are −4.2, −3.9, and −4.2 kcal/mol, respectively. DPPH as a ligand can form 2 hydrogen bonds with His5 and Pi bonds with Met7 on YPLPR (Figure 5B1,B2). The amino acids of DPPH and AFEPLR are mainly bound by Pi bonds (Leu5) and hydrophobic interactions (Ala1, Phe2, and Glu3) (Figure 5C1,C2). DPPH forms a hydrogen bond with Asp2 on NDPF with a bond distance of 2.5 Å and a Pi bond with Phe4 (Figure 5D1,D2). In summary, the results indicated that amino acid residues belonging to YPLPR, AFEPLR, and NDPF could bind to ABTS and DPPH free radicals through hydrogen bonds, π bonds, or hydrophobic interactions, which made these peptides have good ABTS or DPPH free radical scavenging activity.

#### 3.8.3. The Molecular Docking of Bioactive Peptides with Insulin Signaling Pathway

**Figure 6:**
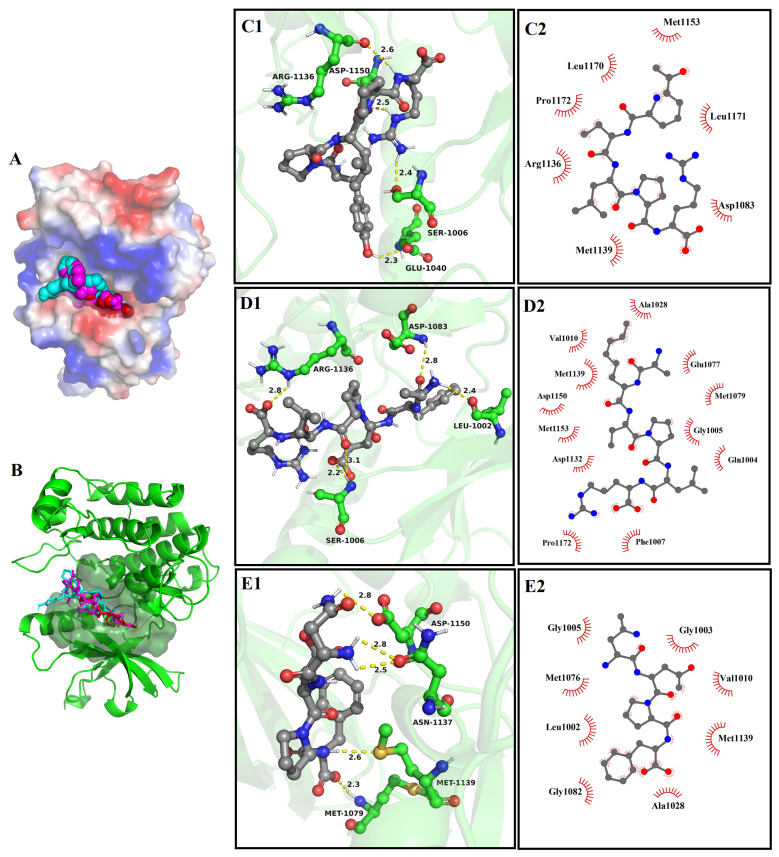
Molecular docking results of YPLPR, AFEPLR, and NDPF with PIRTK protein (PDB Code: 1IR3). The 3D structural surface images of three peptide with receptor protein (**A**); active site binding of three peptide with PIRTK protein (**B**), where YPLPR is in blue, AFEPLR in purple, and NDPF in red. The 3D images of the hydrogen bonding of YPLPR (**C1**), AFEPLR (**D1**), and NDPF (**E1**) with the amino acid residues of PIRTK protein (The yellow dotted line represents the hydrogen bond distance.), and the 2D images of the hydrophobic interaction of YPLPR (**C2**), AFEPLR (**D2**), and NDPF (**E2**) with the amino acid residues of PIRTK protein.

**Figure 7:**
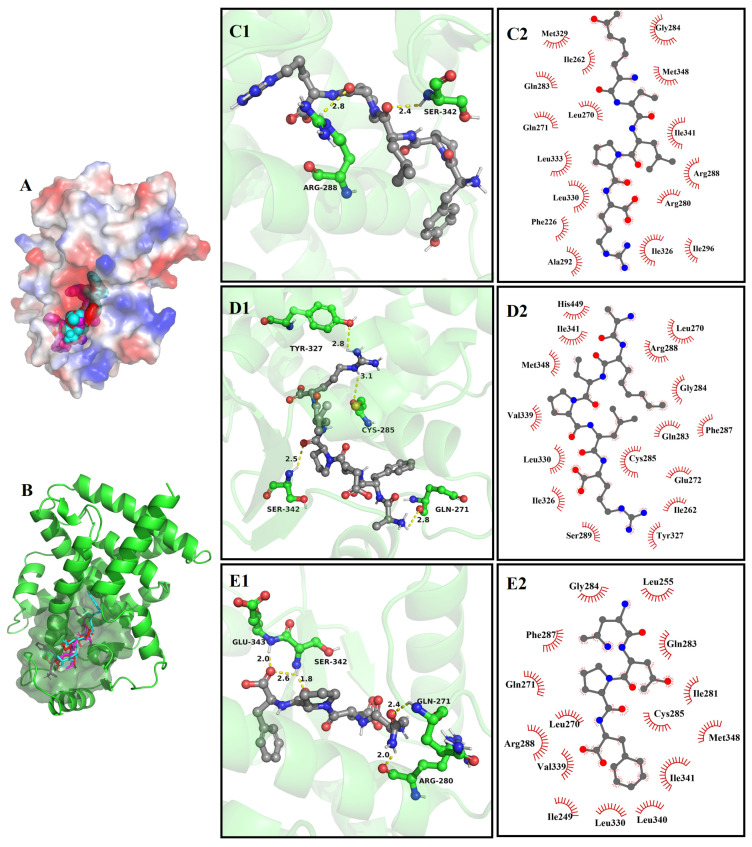
Molecular docking results of YPLPR, AFEPLR, and NDPF with PPARγ (PDB Code: 2PRG). The 3D structural surface images of three peptide with PPARγ (**A**); active site binding of three peptide with receptor protein (**B**), where YPLPR is in blue, AFEPLR in purple, and NDPF in red. The 3D images of the hydrogen bonding of YPLPR (**C1**), AFEPLR (**D1**), and NDPF (**E1**) with the amino acid residues of PPARγ (The yellow dotted line represents the hydrogen bond distance.), and the 2D images of the hydrophobic interaction of YPLPR (**C2**), AFEPLR (**D2**), and NDPF (**E2**) with the amino acid residues of PPARγ.

The molecular docking results between YPLPR, AFEPLR, and NDPF and PIRTK protein and PPARγ are shown in Figure 6 and Figure 7. Peptides YPLPR, AFEPLR, and NDPF were able to enter the active cavities of PIRTK protein (Figure 6A,B) and PPARγ (Figure 7A,B) and were completely encapsulated in the active pockets of PIRTK proteins and PPARγ, forming relatively stable binding conformations. For PIRTK protein, the binding energies of YPLPR, AFEPLR, and NDPF to PIRTK protein were −7.2, −7.7, and −7.3 kcal/mol, respectively. YPLPR formed 4 hydrogen bonds with Ser 1006, Glu 1040, Arg 1136, and Asp 1150 on PIRTK protein (Figure 6C1), AFEPLR formed 5 hydrogen bonds with Leu 1002, Ser 1006, Asp 1083, and Arg 1136 on PIRTK protein (Figure 6D1), and NDPF formed 5 hydrogen bonds with Met 1079, Asn 1137, Met 1139, and Asp 1150 on PIRTK protein (Figure 6E1). And their average bond lengths were 2.45 Å, 2.66 Å, and 2.6 Å, respectively. Meanwhile, peptides YPLPR, AFEPLR, and NDPF were also hydrophobic with 7, 12, and 8 amino acid residues on the PIRTK protein, respectively (Figure 6C2,D2,E2). For PPARγ, the binding energies of peptides YPLPR, AFEPLR, and NDPF to PPARγ were −9.2, −8.8, and −8.5 kcal/mol, respectively. YPLPR was able to form hydrogen bonds with amino acid residues Arg288 and Ser 342 on PPARγ (Figure 7C1), AFEPLR was able to form hydrogen bonds with amino acid residues Gln 271, Cys 285, Tyr 327, and Ser 342 on PPARγ (Figure 7D1), and NDPF can form 5 hydrogen bonds with Gln 271, Arg 280, Ser 342, and Glu 343 on PPARγ (Figure 7E1). Peptides YPLPR, AFEPLR, and NDPF also have hydrophobic interactions with 16, 16, and 15 amino acid residues on PPARγ, respectively (Figure 7C2,D2,E2). These strong hydrogen bonding and hydrophobic interactions both stabilize the binding of YPLPR, AFEPLR, and NDPF to PIRTK protein and PPARγ, thus potentially inhibiting the activity of PIRTK protein and PPARγ.

## 4. Conclusions

The results showed that a total of 20 peptides with ALC scores > 85% were identified in the distillers’ grains peptides obtained by alkaline protease enzymatic digestion, and the molecular weights were concentrated between 438 and 860 Da. The distillers’ grains peptides had good α-glucosidase inhibitory activity and antioxidant activity. Bioinformatics analysis showed that peptides YPLPR, AFEPLR, and NDPF were the main potentially bioactive peptides in distillers’ grains peptides and the three peptides had good α-glucosidase inhibitory activity, insulin resistance-improving and antioxidant activities. And peptides YPLPR, AFEPLR, and NDPF may exhibit their bioactives by stably binding to α-glucosidase, ABTS, and DPPH radicals, as well as relevant targets of the insulin signaling pathway, through strong hydrogen bonding and van der Waals forces. Generally speaking, these results obtained in the present work may offer a potential natural resource for alleviating oxidative stress damage and controlling blood glucose in diabetes, thereby enhancing the utilization of distillers’ grains peptides and augmenting their economic value. However, it should be noted that the methods used in the current study were all in vitro, which may differ from the real environment of the human body; the bioavailability, intestinal absorption, and bioactivities of these peptides need to be further investigated in vivo in the future.

## Figures and Tables

**Table 1:** Peptides in distillers’ grains protein hydrolysates.

Peptides	ALC (%)	RT (min)	Mass (Da)	Local Confidence (%)
ELELLE	96	2.31	744.3905	99 97 98 94 95 98
RFDR	96	2.2	592.3081	95 96 97 98
PDVGHPM	94	14.64	751.3323	89 97 97 91 96 94 96
YPLPR	92	15.28	644.3646	94 97 96 89 83
VLEPR	91	7.08	612.3595	88 98 97 85 90
AFEPLR	91	15.98	731.3966	90 97 98 85 92 87
FEEL	91	17.76	536.2482	86 93 96 89
WNVN	88	14.76	531.2441	85 79 94 94
EEEF	88	13.81	552.2067	72 94 98 89
FEPLR	88	14.89	660.3595	86 98 81 91 82
WLDY	87	18.69	595.2642	87 89 88 87
ERR	87	1.04	459.2554	96 85 81
FEPL	87	18.25	504.2584	86 94 79 90
LDFEPR	87	17.02	775.3864	88 91 90 96 76 79
FDGVLRGP	86	17	859.4551	89 96 90 95 89 74 79 79
YAGE	86	2.81	438.175	86 85 77 95
NDPF	85	15.77	491.2016	84 87 84 88
RVLEPR	85	4.98	768.4606	82 88 90 95 79 77
WTVN	85	15.35	518.2489	80 82 91 88
WNLN	85	17.09	545.2598	85 77 93 85

RT: retention time.

**Table 2:** Peptides with the main potentially bioactive selected for in silico analyses.

Peptides	Tag Length	Peptide Ranker Score	Affinity (kcal/mol)	Estimated Toxicity	Estimated Solubility
RFDR	4	0.6986	−8.7	Non-toxin	Good
PDVGHPM	7	0.6039	−8.6	Non-toxin	Good
YPLPR	5	0.7972	−10.7	Non-toxin	Good
AFEPLR	6	0.7571	−9.6	Non-toxin	Good
FEPLR	5	0.7106	−9.0	Non-toxin	Good
WLDY	4	0.7981	−8.6	Non-toxin	Poor
FEPL	4	0.7531	−8.8	Non-toxin	Good
FDGVLRGP	8	0.6470	−8.7	Non-toxin	Good
NDPF	4	0.8886	−9.1	Non-toxin	Good
WNLN	4	0.6784	−9.2	Non-toxin	Poor
WLRL (Positive control)	4	0.9210	−9.1	Non-toxin	Poor

**Table 3:** The molecular docking results of YPLPR, AFEPLR, and NDPF with α-glucosidase.

	YPLPR	AFEPLR	NDPF
Affinity (kcal/mol)	−10.7	−9.6	−9.1
Number of hydrogen interactions	9	11	3
Amino acid residues involved in hydrogen bonds	Asp 215, Ser 240, Ser 241, Asp 242, Glu 277, Gln 279, Phe 314, Arg 315, Asp 352	Lys 156, Ser 240, Ser 241, His 280, Pro 312, Arg 315, Gln 353	Lys 156, Tyr 158, Asp 242
Number of hydrophobic interactions	10	15	8
Amino acid residues participated in van der waals	Lys 156, Tyr 158, Phe 178, Val 216, Phe 303, Pro 312, Leu 313, Phe 314, Arg 315, Gln 353	Ser 157, Tyr 158, Phe 159, Phe 178, Val 216, Gln 239, Glu 277, His 280, Phe 303, Pro 312, Leu 313, Arg 315, Asp 352, Glu 411, Arg 442	Tyr 158, Phe 178, Glu277, Gln 279, Phe 303, Pro 312, Arg 315, Arg 442

## Data Availability

The original contributions presented in the study are included in the article/Supplementary Materials, further inquiries can be directed to the corresponding author due to privacy.

## Supplementary Materials

The following supporting information can be downloaded at: https://www.mdpi.com/article/10.3390/nu16091279/s1. Methods: The methods used in this experiment were described in detail.
