# Supplementary material for: Identification of Novel Peptides in Distillers’ Grains as Antioxidants, α-Glucosidase Inhibitors, and Insulin Sensitizers: In Silico and In Vitro Evaluation
Source: Nutrients. 2024 Apr 25;16(9):1279. doi: 10.3390/nu16091279 (PMC11085682; doi:10.3390/nu16091279)
Supplement: Supplementary file 1 [file nutrients-16-01279-s001.zip › nutrients-2939916-supplementary.pdf]

Supporting Information

Methods

1. Sample preparation

Initially, 350.0 g of distillers' grains powder was mixed with distilled water at a proportion of 1:6 (w/v), and the pH was regulated to 9.0 by 1.0 mol/L NaOH. The mixture was mixed at 57°C for 2 hours and extracted by ultrasonication for 30 minutes (at a power of 300 W), which was then centrifuged for 15 minutes and 3000×g (4°C). The protein was deposited by lowering the pH of the supernate to 4.0 using 1.0 mol/L HCl, followed by another centrifugation and 3000×g for 15 minutes (4℃). The resulting precipitate, referred to as distillers' grains proteins, was subsequently freeze-dried and stored (-30°C) [1]. The distillers' grains proteins were subjected to enzymatic digestion following the procedure depicted by Xie et al. (2022) [2]. Initially, the lyophilized distillers' grains protein was solubilized in distilled water (50.0 mg/mL). Alkaline protease was added at a dosage of 5.0% (E/S, w/w). The solution was stirred at a pH of 8.0 and maintained at 55°C for 2.5 hours. To terminate the enzymatic reaction, the solution was heated in a boiling water bath for 10 minutes and subsequently centrifuged 6000×g for 10 minutes (4°C). The resulting supernatant was then freeze-dried and stored (-30 °C).

2. Composition analysis of peptide by UHPLC-ESI-HRMS/MS

According to Tian et al. (2022) method (slightly modified), the mass spectrometry pa-rameters were set. This analysis was conducted in positive ion mode and utilized an Agilent Zorbax SB-C18 column (1.7 μm, 2.1 mm × 100 mm) with specific mobile phases and conditions. Eluent A (ultrapure water solution of 0.1% formic acid) and eluent B (acetonitrile solution of 0.1% formic acid) constituted the mobile phase, the flow velocity was 0.2 mL/min. A 3.0 μL injection volume was utilized, and the column temperature was maintained at 30 °C. The gradient program was as follows: 0 to 3 minutes, 5.0% B; 3 to 10 minutes, 5.0-10.0% B; 10 to 15 minutes, 10.0-20.0% B; 15 to 20 minutes, 20.0-95.0% B; 20 to 22 minutes, 95.0-5.0% B; 22 to 25 minutes, 5.0% B. The scanning range covered 150–2000 m/z [3].

3. Determination of *α*-glucosidase inhibitory effect

The inhibitory effect on *α*-glucosidase activity of the protein hydrolysates from distillers' grains or three selected peptides was assessed following the methodology outlined by Zhang et al. (2018) with slight modifications [4]. Samples with varying concentrations were dissolved in 10.0 μL of PBS at pH 6.8. The *α*-glucosidase solution was also dissolved in 10.0 μL of PBS at a concentration of 7 U/mL. These solutions were mixed with 100.0 μL of PBS and then incubated at 37°C for 15 minutes. Subsequently, 10.0 μL of 2.5 mmol/L pNPG solution was added and incubated at 37 °C for 30 minutes. Afterward, 60.0 μL of 20 mmol/L NaCO3 solution was introduced to the mixed solution. The absorbance of the resulting reaction mixture was measured at a wavelength

of 405 nm using an enzyme-labeled instrument. Different control groups were utilized for comparison to determine the α-glucosidase inhibition rate for the samples: α-Glucosidase Inhibition Rate (%) = [1 - (A_{sample} - $A_{sample\ blank}$) / ($A_{control}$ - $A_{control\ blank}$)] × 100 (1). In this formula, "A" represents the absorbance value.

4. **Assessment of scavenging capacity against DPPH and ABTS radicals**

The antioxidant properties of vinasse hydrolysates or three screening peptides were evaluated by DPPH and ABTS free radical scavenging experiments according to the method of Sun et al. (2015) [5]. For DPPH analysis, 50.0 μL of sample solution was combined with 200.0 μL of DPPH (0.1 mmol/L), and the reaction mixture was incubated in darkness for 30 minutes. The absorbance was then measured at 517 nm. For ABTS analysis, 5 μL of ABTS solution (7 mmol/L) and 88.0 μL of potassium persulfate solution (40 mmol/L) were mixed and reacted in darkness for 12-16 hours. The ABTS solution was diluted with ethanol, and the absorbance was 0.70 ± 0.02 at 745 nm. Subsequently, 50.0 μL of each sample was added to 400.0 μL of ABTS solution and incubated for 10 minutes before measurement. The absorbance was measured at 745 nm. The sample blank group comprised 200.0 μL of methanol (DPPH) or 400.0 μL of ethanol (ABTS), the control group comprised 50.0 μL of distilled water, and the blank control group was a combination of 50.0 μL of distilled water and 200.0 μL of methanol (DPPH) or ethanol (ABTS). The following formulas were used to determine the DPPH and ABTS radical scavenging rates of samples: Free Radical Scavenging Rate (%) = [1 - (A_{sample} -

$A_{sample\ blank}$) / ($A_{control}$ - $A_{control\ blank}$)] × 100 (2). In this formula, "A" represents the absorbance value.

5. In silico screening of main bioactive peptides in distillers' grains protein hydrolysates

The detailed method of studying the interaction between distiller's grains protein hy-drolysate polypeptide and α -glucosidase by molecular docking technology was noted in the annex. For the pretreatment of peptide, refer to O 'Boyle et al. (2011) [6]. Initially, the amino acid sequences of peptides were converted into Simplified Molecular Input Line Entry Specification (SMILES) strings using PepSMI (https://www.novoprolabs.com/tools/convert-peptide-to-smiles-string). Furthermore, the software Openbabel 3.1.1 was used to transform multiple SMILES strings into 3D molecular structures. These peptide structures were refined through the addition of polar atomic hydrogen, Gasteiger charges, and optimization using the MMFF94 field.

6. Determination of insulin-resistance HepG2 cells on bioactive peptides

HepG2 cells at logarithmic growth stage were vaccinated in 96-well plates with 200 μL (5×10^4 cells/mL) and cultured for 24 h. After aspirating the medium, 200 μL of normal DMEM medium was added to the blank group, and 25.0 μmol/L of insulin was added to the sample, positive control and model groups for 24 h. The medium was aspirated and washed twice with PBS, and each group was incubated with serum-free medium (without phenol red) on dissolved samples for 24 h, where the sample group peptide YPLPR, AFEPLR

and NDPF concentrations were 1.0 mmol/L, and the positive control rosiglitazone concentration was 20.0 μmol/L. At the end of the incubation, the glucose content in the culture medium was measured using the glucose oxidase-peroxidase method kit by Nanjing Jiancheng Biological Co., Ltd., and the glucose content was calibrated by measuring the cell activity using the MTT method [7,8].

7. Evaluation of scavenging capacity against intracellular ROS

HepG2 cells (2.0 mL/well, 1×10^4 cells/well) were inoculated in 6-well plate and cultured overnight. VC (10.0 μmol/L) or peptides YPLPR, AFEPLR, and NDPF (1.0 mmol/L) were added to each well, and the cells were incubated for an additional 24 hours. Following the incubation, the culture medium was aspirated, and the cells were washed thoroughly with PBS twice. Subsequently, cells in all experimental groups, except for the control group, were exposed to 1.2 mmol/L H_2O_2 for seven hours. The cells were then harvested, washed twice with cold PBS, and incubated with a DCFH-DA probe solution (10.0 μmol/L) in the dark at 37°C for 20 minutes. After incubation, the cells were rinsed twice with culture medium devoid of FBS and analyzed using a Millipore flow cytometer (Guava easy Cyte 6-2L, Billerica, MA) within 30 minutes [9].

References:

1. Wang, R.; Zhao, H.; Pan, X.; Orfila, C.; Lu, W.; Ma, Y. Preparation of Bioactive Peptides with Antidiabetic, Antihypertensive, and Antioxidant

Activities and Identification of A-glucosidase Inhibitory Peptides from Soy Protein. Food Sci Nutr 2019, 7, 1848–1856, doi:10.1002/fsn3.1038.

2. XIE, Y.; Wang, J.; Li, Z.; Luan, Y.; Li, M.; Peng, X.; Xiao, S.; Zhang, S. Damage Prevention Effect of Milk-Derived Peptides on UVB Irradiated Human Foreskin Fibroblasts and Regulation of Photoaging Related Indicators. Food Research International 2022, 161, 111798, doi:10.1016/j.foodres.2022.111798.
3. Wenhui, T.; Shumin, H.; Yongliang, Z.; Liping, S.; Hua, Y. Identification of in Vitro Angiotensin-converting Enzyme and Dipeptidyl Peptidase <scp>IV</Scp> Inhibitory Peptides from Draft Beer by Virtual Screening and Molecular Docking. J Sci Food Agric 2022, 102, 1085–1094, doi:10.1002/jsfa.11445.
4. Zhang, X.; Jia, Y.; Ma, Y.; Cheng, G.; Cai, S. Phenolic Composition, Antioxidant Properties, and Inhibition toward Digestive Enzymes with Molecular Docking Analysis of Different Fractions from Prinsepia Utilis Royle Fruits. Molecules 2018, 23, 3373, doi:10.3390/molecules23123373.
5. Sun, D.; Huang, S.; Cai, S.; Cao, J.; Han, P. Digestion Property and Synergistic Effect on Biological Activity of Purple Rice (Oryza Sativa L.) Anthocyanins Subjected to a Simulated Gastrointestinal Digestion in Vitro. Food Research International 2015, 78, 114–123, doi:10.1016/j.foodres.2015.10.029.

6. O'Boyle, N.M.; Banck, M.; James, C.A.; Morley, C.; Vandermeersch, T.; Hutchison, G.R. Open Babel: An Open Chemical Toolbox. J Cheminform 2011, 3, 33, doi:10.1186/1758-2946-3-33.
7. Zhou, M.; Bu, T.; Zheng, J.; Liu, L.; Yu, S.; Li, S.; Wu, J. Peptides in Brewed Wines: Formation, Structure, and Function. J Agric Food Chem 2021, 69, 2647–2657, doi:10.1021/ acs.jafc. 1c00452.
8. Zhu, D.; Wang, Y.; Du, Q.; Liu, Z.; Liu, X. Cichoric Acid Reverses Insulin Resistance and Suppresses Inflammatory Responses in the Glucosamine-Induced HepG2 Cells. J Agric Food Chem 2015, 63, 10903–10913, doi:10.1021/acs. jafc. 5b04533.
9. Liu, X.; Wang, O.; Zhou, J.; Cai, S. Different Phenolic Extracts of Oil Palm Fruits and Caffeic Acid Prevent Palmitic Acid-Induced Lipotoxicity in HepG2 Cells via Improving Mitochondrial Function. J Food Qual 2020, 2020, 1–12, doi:10.1155/2020/8827707.
